# The importance of psoas muscle on low back pain: a single-center study on lumbar spine MRI

**DOI:** 10.1016/j.xnsj.2024.100326

**Published:** 2024-05-01

**Authors:** Carlo A. Mallio, Fabrizio Russo, Gianluca Vadalà, Rocco Papalia, Matteo Pileri, Valeria Mancuso, Caterina Bernetti, Manuel Volpecina, Gianfranco Di Gennaro, Bruno Beomonte Zobel, Vincenzo Denaro

**Affiliations:** a Fondazione Policlinico Universitario Campus Bio-Medico, Via Alvaro del Portillo, 200, 00128 Roma, Italy; b Research Unit of Radiology, Department of Medicine and Surgery, Università Campus Bio-Medico di Roma, Via Alvaro del Portillo, 21, 00128 Roma, Italy; c Research Unit of Orthopaedic and Trauma Surgery, Department of Medicine and Surgery, Università Campus Bio-Medico di Roma, Via Alvaro del Portillo, 21, 00128 Roma, Italy; d Department of Health Sciences, Chair of Medical Statistics, University of Catanzaro Magna Græcia, Viale Europa, 88100 Catanzaro, Italy

**Keywords:** Low back pain, MRI imaging, Muscle cross sectional area (csa), Intramuscular fat infiltration, Visual Analogue Scale (VAS), Spine, Obesity

## Abstract

**Background:**

Low back pain (LBP) is the most frequent indication to magnetic resonance imaging (MRI) examinations of the lumbosacral spine. The individual role of soft tissues, including muscles, on LBP is not fully understood and the contribution of each MRI-derived parameter of soft tissues status on the intensity of LBP has not been investigated in detail.

**Methods:**

The study design was observational retrospective, single center carried out at a University Hospital. Images were acquired using a using a 1.5 Tesla scanner. Patients completed a symptom questionnaire and rated their pain intensity using the Visual Analogue Scale (VAS). The VAS scores ​​were categorized as mild, moderate, and severe using cutoff values of 3.8 and 5.7, based on the literature. Biometric data, including weight and height, were also recorded to calculate the body mass index (BMI). The ratios between intramuscular fat infiltration and net muscle area were also calculated. Patient sample included 94 patients with LBP underwent MRI of the lumbosacral spine.

**Results:**

The stepwise analysis revealed that increasing psoas net area was associated with lower VAS levels (odds ratio [OR]: 0.94: 95% confidence interval [CI]: 0.90–0.98; p=.005), and an increase of one square centimeter of total psoas area resulted in a greater probability of reporting a mild (+1.21%; 95% CI: 0.37, 2.05%) or a moderate VAS (+0.40%; 95% CI: -0.02, 0.82%), Furthermore, a more severe VAS was associated with a higher BMI (OR: 1.13; 95% CI: 1.00–1.27).

**Conclusion:**

Our study demonstrates a relationship between LBP and MRI parameters of paravertebral and psoas muscles status. The psoas muscle is extremely important for spine stabilization and is linked to clinical symptoms of patients affected by LBP. These findings could contribute to future studies and improve treatment options in patients with LBP, possibly reducing the impact on disability, quality of life and socioeconomical burden.

## Introduction

Low back pain (LBP) is a highly prevalent symptom, also affecting the working-age population [[1], [2], [3]]. Indeed, LBP ranks among the leading causes of work absences and determines significant social and health costs burden associated with therapy and rehabilitation [4].

Degenerative disk disease is a natural consequence of aging and can be associated to LBP [5]. As a function of age increase, intervertebral discs gradually lose their flexibility, elasticity and capacity to provide cushioning [6].

Magnetic resonance imaging (MRI) is an excellent modality to investigate the soft tissues and the bone, including patients with lumbosacral diseases [[1], [2], [3]].

Despite the accuracy of imaging techniques, such as MRI, the root cause of pain cannot always be determined. In fact, several studies have demonstrated that disk degeneration can be present in many asymptomatic individuals, particularly among the elderly [[7], [8], [9], [10]]. Conversely, only minimal degenerative disk changes are often found in patients with LBP and related disability [11].

Studies aimed to identify the causes of LBP primarily focused on bones, discs, and joint components [12]. However, research attention on the topic has been recently focused on the role of paraspinal soft tissues, including the muscular system. In this context, the individual role of soft tissues on LBP is not fully understood and the contribution of each MRI-derived parameter of soft tissues status on the intensity of LBP has not been investigated in detail.

This study aimed primarily to investigate the potential relationship between LBP and MRI parameters indicative of muscle quantity and quality of paravertebral and psoas muscles.

## Methods

This research study was approved by the local ethical committee and complied with the 1964 Helsinki declaration and its later amendments or comparable ethical standards.

### Study design and population

This retrospective study enrolled patients with LBP, who underwent orthopedic clinical assessment and MRI examination of the lumbar spine at Fondazione Policlinico Campus Bio-Medico, between November 2019 and March 2022.

Inclusion criteria were symptomatic chronic LBP due to degenerative disk disease, workers (has worked at least 2 months, even if not continuously, in the last 6 months); age between 18 and 65 years; signed informed consent. Exclusion criteria were non-workers; age<18 or >65; LBP due to spondylodiscitis, traumatic or neoplastic causes; congenital or acquired diseases leading to spine deformities.

All patients underwent lumbar spine MRI within one month after their clinical orthopedic evaluation, thus, they all had LBP at the time of the MRI execution.

### MRI protocol

MRI scans were conducted using a 1.5 Tesla Magnetom Aera scanner (Siemens, Erlangen, Germany). The patients were properly positioned, and the following sequences were acquired:

• Sagittal T1-weighted Turbo Spin Echo (TSE) imaging from D12 to the sacrum (TR=368 ms, TE=8,3 ms, Dist. factor=20%, Phase enc. dir.=*H* >> *F*, FoV read=320 mm, FoV phase=100,0%, voxel size=1.0×1.0×3.0 mm).
• Sagittal T2-weighted TSE imaging from D12 to the sacrum (TR=3870 ms, TE=107 ms, Dist. factor=20%, Phase enc. dir.=*H* >> *F*, FoV read=320 mm, FoV phase=100,0%, voxel size=0.8×0.8×3.0 mm).
• Sagittal T2-weighted DIXON fat-suppressed sequence from D12 to the sacrum (TR=4000 ms, TE=92 ms, Dist. factor=20%, Phase enc. dir.=*H* >> *F*, FoV read=320 mm, FoV phase=100,0%, voxel size=0.8×0.8×3.0 mm).
• Axial T2-weighted TSE imaging from L1 to S1 (TR=4840 ms, TE=102 ms, Dist. factor=10%, Phase enc. dir.=*A* >> *P*, FoV read=240 mm, FoV phase=84.4%, voxel size=0.8×0.8×3.0 mm).

### Outcome measures

The OsiriX™ software (version 3.8.1, Pixmeo, Geneva, Switzerland) was used to measure, on the axial T2 images, the cross-sectional area (CSA) of ​​the paravertebral and psoas muscles and the intramuscular fat infiltration. Thus, we calculated both total CSA and net area (i.e., muscular area only without including intramuscular fat infiltration) of each evaluated muscle.

Images were segmented manually at the level of L4–L5, according to the methods of previously published studies [[13], [14], [15]]. The maximum thickness of the posterior subcutaneous adipose tissue in the lumbar region was evaluated on the same slice. The presence or absence of posterior subcutaneous lumbar spine edema was recorded for each examination. Moreover, data were gathered on disk degeneration from L1 to S1 and graded according to the Pfirrmann classification.

**Fig. 1:**
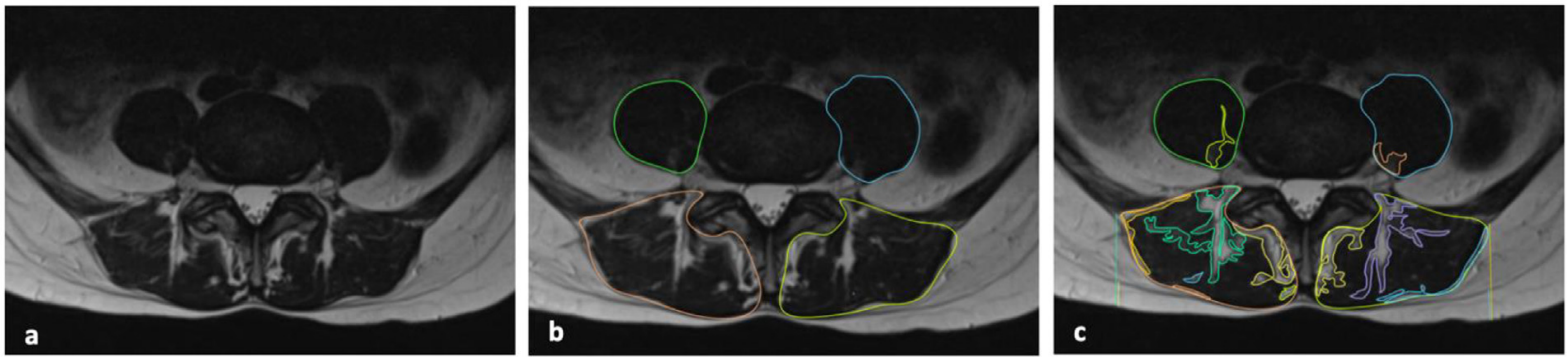
(**A**) Axial T2-weighted sequence at the L4–L5 intervertebral disk level. Native image. (**B**) The cross-sectional total muscle area of the paravertebral and psoas muscles was calculated using fine manual segmentation with polygonal points. (**C**) The intramuscular adipose infiltration and the maximum thickness of the posterior subcutaneous adipose tissue were measured by drawing a line from the lateral margin of the paraspinal muscle to the dermal boundary.

**Fig. 2:**
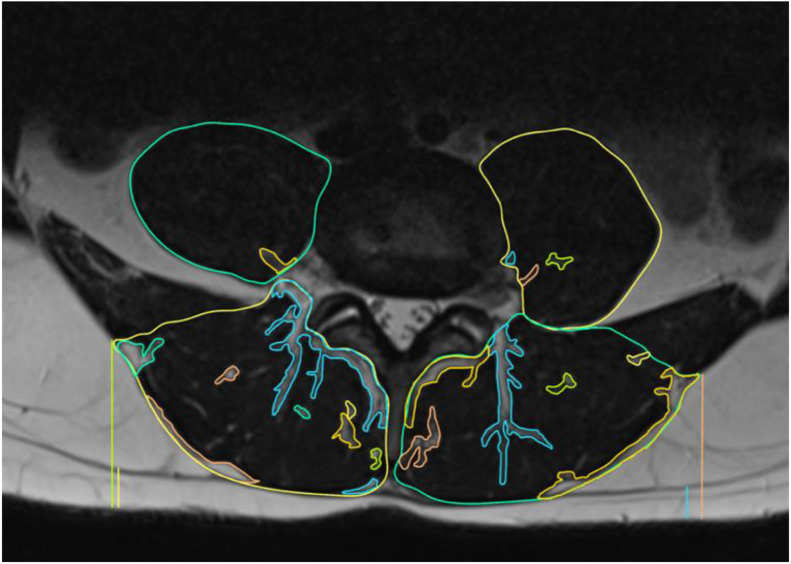
The image shows good muscle trophism and mild intramuscular adipose infiltration of the paraspinal muscles and psoas muscles.

**Fig. 3:**
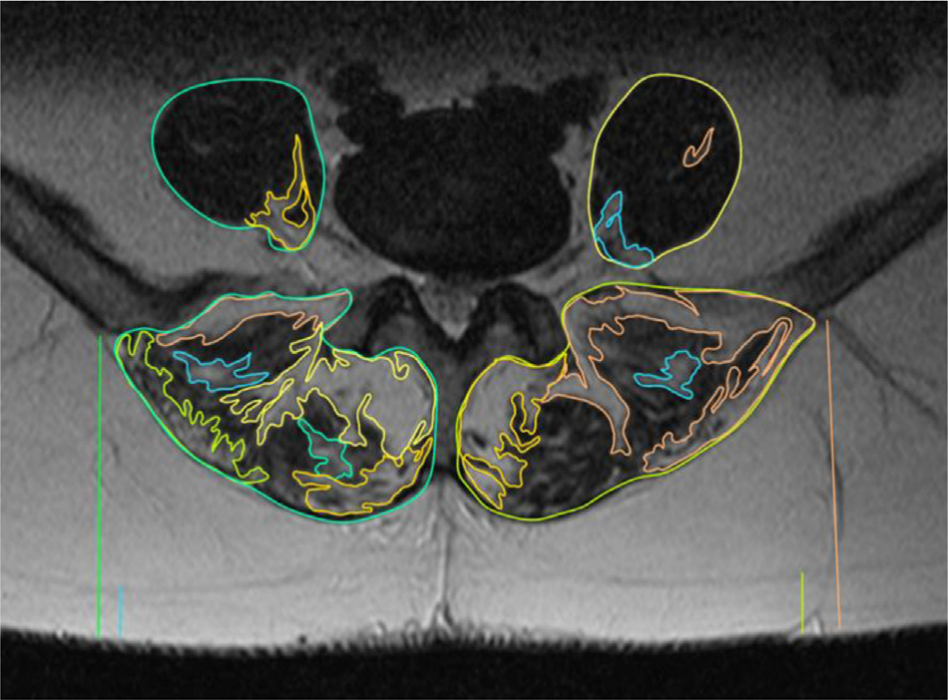
Patient n. 3. Evidence of sustained muscle hypotrophy together with significant intramuscular adipose infiltration of the paraspinal muscles.

Segmentations and all images analysis and evaluations were performed by consensus of two Radiologists (C.A.M., 12 years of experience, and V.M., 5 years of experience) (Figs. 1–3).

Patients answered a questionnaire regarding their symptoms and rated their pain intensity using the Visual Analogue Scale (VAS). The VAS scores ​​were categorized as mild (VAS≤3.8), moderate (3.8<VAS≤5.7) and severe (VAS>5.7), as previously described [16].

Biometric data, including weight and height, were also collected and body mass index (BMI) was calculated. Ratios between net muscle area and intramuscular fat infiltration and the psoas and paravertebral muscles' total area (left and right) were subsequently calculated.

### Statistical analysis

Gaussian continuous variables were described using the mean and standard deviation. In cases of skewness, the median and interquartile ranges were considered. Counts and percentages were used for categorical variables. The normality distribution of continuous variables was assessed using the Shapiro-Wilk test. Bivariate analyses were performed to compare the variables across the three levels of VAS (i.e., low, medium, high). More precisely, one-way ANOVA was used for normally distributed continuous variables, while the Kruskal–Wallis test for variables with skewed distributions. Categorical variables were analyzed using the 3×2 chi-squared or Fisher's exact test for cells with low counts (<5). To identify the possible predictors of the VAS category, the effect of each variable was adjusted for the value of the other variables through an exploratory stepwise-backward ordinal regression model. Variables with a p-value greater than .2 were removed from the model. Finally, the marginal effects of the selected predictors by the stepwise model on the patient's probabilities of belonging to each of the three VAS score categories were calculated. The study had exploratory and hypothesis-generating objectives. No a priori power analysis was conducted. Significance level was set at 5%. The statistical analyses were performed using STATA (version 16.0, StataCorp, www.stata.com).

## Results

### Sample characteristics

We observed 94 patients, of which 55 (58.51%) were men and 39 (41.5%) were women. The median age was 48.5 years [interquartile range (IQR: 42–55)]. Moreover, the median BMI was 24.4 unit Kg/m^2^ (IQR: 22.4–26.64) and 30 (31.9%) patients suffered from posterior subcutaneous lumbar spine edema. A mild VAS-score was reported by 25 (26.6%) patients, whereas a moderate and severe VAS score was reported by 19 (20.2%) and 50 (53.2%) patients, respectively. The median age in mild, moderate, and severe VAS groups was 47 (IQR: 43–56), 51 (IQR: 42–55) and 47 (IQR: 42–54) years respectively, and no statistically significant difference emerged (p=.697). Furthermore, the three groups of patients (mild-moderate-severe VAS) were not significantly different in terms of BMI (mild VAS: 23.25, IQR: 22.04–25.77; moderate VAS: 25.42, IQR: 22.60–27.99; severe VAS: 24.40, IQR: 22.40–27.70. p=.724) and total Pfirmann Score (mild VAS: 14.24, standard deviation [sd]: 1.56; moderate VAS: 14.32, sd: 2.43; severe VAS: 14.14, sd: 1.84. p=.937). A summary of all patients’ characteristics and significance of between-groups differences, including results about muscular status, are reported in Table 1.

**Table 1:** Overall and VAS-subgrouped characteristics of study patients.

	Overall		Mild VAS		Moderate VAS		Severe VAS		p
**Sex**	Men: 55 (58.51%)		Men: 17 (68.00%)		Men: 13 (68.42%)		Men: 25 (50.00%)		.203
	Women: 39 (41.49%)		Women: 8 (32.00%)		Women: 6 (31.58%)		Women: 25 (50.00%)		
Age*	48.50	(42.00 - 55.00)	47.00	(43.00 - 56.00)	51.00	(42.00 - 55.00)	47.00	(42.00 - 54.00)	.697
CSA psoas*	31.48	(23.02 - 40.58)	35.91	(23.69 - 42.56)	38.86	(23.79 - 42.32)	26.79	(22.25 - 37.30)	.164
CSA paravertebral*	53.51	(47.02 - 61.93)	52.11	(46.37 - 58.43)	57.62	(47.92 - 62.50)	52.41	(46.52 - 61.93)	.523
Psoas net area*	29.62	(20.78 - 38.22)	33.89	(22.21 - 42.04)	36.54	(22.30 - 40.74)	24.00	(20.58 - 35.40)	.092
**Paravertebral net area**⁎⁎	37.77	(9.39)	37.55	(8.50)	39.85	(8.42)	37.09	(10.19)	.551
Psoas fat*	1.77	(1.18 - 2.52)	1.57	(1.15 - 2.18)	1.89	(1.16 - 3.08)	2.00	(1.25 - 2.52)	.838
Paravertebral fat*	15.79	(12.97 - 19.12)	14.86	(12.63 - 16.33)	16.70	(13.39 - 19.12)	16.12	(13.26 - 20.13)	.419
Total fat*	9.92	(8.25 - 11.41)	9.78	(8.24 - 10.85)	10.38	(8.29 - 12.69)	10.01	(8.25 - 11.60)	.518
Visceral fat*	7.58	(6.28 - 9.20)	7.45	(6.09 - 8.60)	8.30	(6.54 - 9.76)	7.49	(6.39 - 9.20)	.591
Superficial fat*	1.99	(1.47 - 2.54)	1.83	(1.51 - 2.16)	2.00	(1.52 - 2.29)	2.16	(1.45 - 2.75)	.215
BMI*	24.40	(22.40 - 26.64)	23.25	(22.04 - 25.77)	25.42	(22.60 - 27.99)	24.40	(22.40 - 27.70)	.724
**Total Pfirmann score**⁎⁎	14.20	(1.89)	14.24	(1.56)	14.32	(2.43)	14.14	(1.84)	.937
Paravertebral area/fat ratio*	2.38	(1.87 - 3.04)	2.66	(2.01 - 3.30)	2.42	(1.98 - 3.51)	2.27	(1.66 - 2.73)	.274
Psoas area/fat ratio*	14.94	(9.76 - 26.60)	16.32	(12.46 - 28.19)	17.40	(9.76 - 35.10)	13.84	(9.11 - 18.98)	.342
**Edema**	Edema: 64 (68.09%)		Edema: 19 (76.00%)		Edema: 11 (57.89%)		Edema: 34 (68.00%)		0.443
	No Edema: 30 (31.91%)		No Edema: 6 (24.00%)		No Edema: 8 (42.11%)		No Edema: 16 (32.00%)		

⁎ Median and interquartile range; statistical comparison by Kruskal–Wallis test.

⁎⁎ Mean and standard deviation; statistical comparison by one-way ANOVA. Between-group sex distributions compared by 2×3 Pearson's Chi-Squared test. **Abbreviations**: Cross Sectional Area (CSA); Body Mass Index (BMI).

### Stepwise analysis

**Fig. 4:**
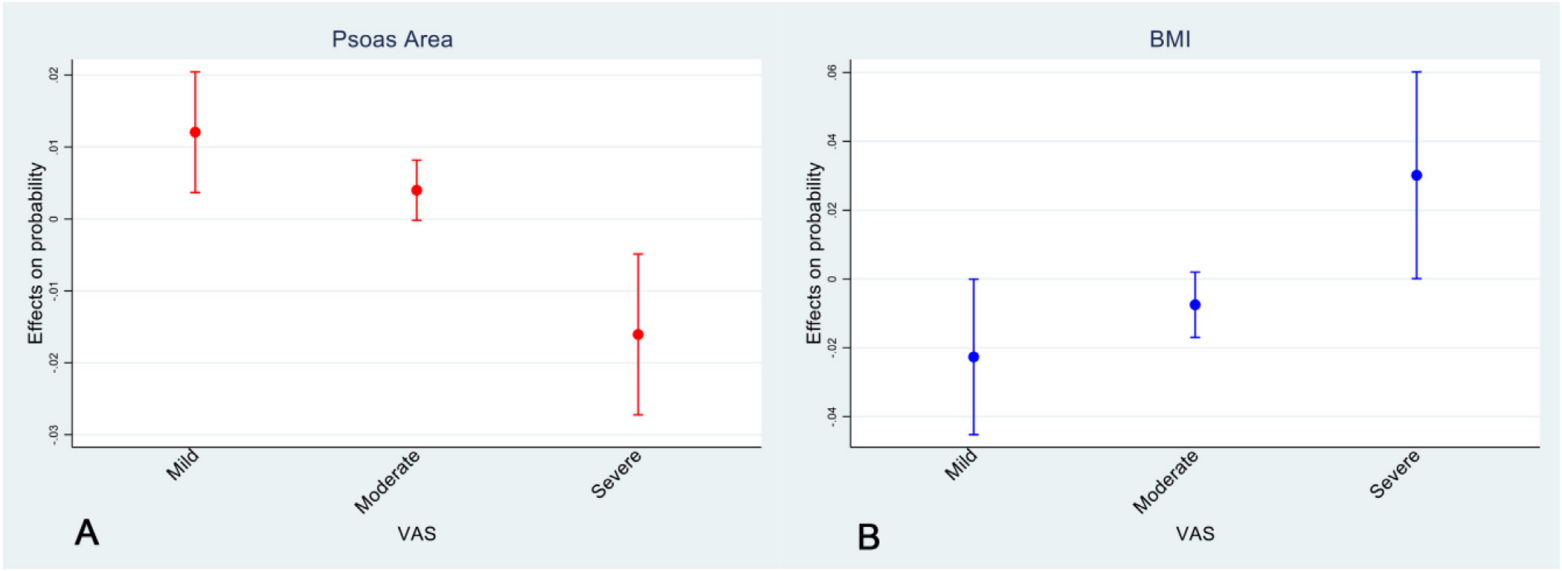
Marginal effects of total psoas area (one square centimeter increase) (A) and BMI (one point increase) (B) on the probability of mild, moderate, and severe VAS score.

When the effect of individual variables was adjusted for the value of the other possible predictors, the stepwise analysis showed that a lower VAS level was observed as the total psoas area increased (odds ratio [OR]: 0.94: 95% confidence interval [CI]: 0.90–0.98) while a more severe VAS was associated with a higher BMI (OR: 1.13; 95% CI: 1.00–1.27). In this regard, the marginal effect analysis (Fig. 4) showed that as one BMI point increases, the probability of a patient suffering from a mild VAS (−2.27%; 95% CI: −4.53, 0.00%) or from a moderate VAS (−0.75%; 95% CI: −0.75, −0.2%) decreases, while the probability of suffering from severe VAS increases (+3.01%; 95% CI: 0.01, 6.02%). Moreover, an increase of one square centimeter of total Psoas area results in an increased probability of reporting a mild (+1.21%; 95% CI: 0.37, 2.05%) or a moderate VAS (+0.40%; 95% CI: −0.02, 0.82%), while the risk of suffering from severe VAS decreases (−1.61%; 95% CI: −2.72, −0.49%). For a summary of the results see Table 2.

**Table 2:** Stepwise-backward ordinal modeling of VAS category.

Outcome: VAS category (mild-moderate-severe)	Odds ratio (95% CI)		p
**Sex**	†		
**Age**	†		
**CSA psoas**	†		
**CSA paravertebral**	†		
**Psoas net area**	0.94	(0.90 - 0.98)	.005
**Paravertebral area**	†		
**Psoas fat**	†		
**Paravertebral fat**	†		
**Total fat**	†		
**Visceral fat**	†		
**Superficial fat**	†		
**BMI**	1.13	(1.00 - 1.27)	.05
**Total Pfirmann score**	†		
**Paravertebral area/fat ratio**	†		
**Psoas area/fat ratio**	†		

Threshold for variables’ elimination: p>.2.

† Variable deleted from the model. **Abbreviations**: Cross Sectional Area (CSA); Body Mass Index (BMI).

## Discussion

Our study demonstrates a relationship between LBP and MRI parameters of psoas muscle status. Indeed, the increased psoas net area was associated with lower VAS in general and one square centimeter of total psoas area resulted in a greater probability of reporting or mild or moderate VAS.

LBP is a highly prevalent symptom among the global population, resulting in significant disability and substantial healthcare costs associated with therapy, rehabilitation, and work absenteeism.

MRI is the best imaging technique to study the lumbosacral spine in patients with LBP. It evaluates the intervertebral disk and, due to its excellent tissue contrast, enables quantitative analysis of soft tissues such as muscles status.

In our study imaging segmentation was performed on sagittal T2 at the level of the L4–L5 intervertebral disk, as previously [[13], [14], [15]]. Several authors found that the total CSA of the paraspinal and psoas muscles is maximal at L4–L5 level, being this an excellent anatomical landmark to obtain a quantitative sample of muscles status [17,18].

The paravertebral and psoas muscles play a crucial role in the dynamic stability of the lumbar spine. Structural alterations in these muscles can compromise spinal stability and lead to further injury and exacerbation of pain symptoms [19]. Consequently, pain hampers patient mobility, by leading to muscle atrophy and initiating a vicious cycle. Therefore, the study of lumbar muscles has garnered significant interest in investigating LBP.

Many authors have demonstrated that lumbar muscle degeneration, characterized by a reduction in muscle size and increased intramuscular fat deposits [20], is a common feature of LBP.

In our study the intensity of LBP quantitatively estimated with the VAS, showed a significant relationship with psoas muscle status as evaluated on MRI images. We evaluated this relationship in a group of working age patients affected by LBP, using a quantitative approach both for LBP and for MRI parameters and achieving results with the statistical stepwise analysis.

The findings the present study revealed that lower pain intensity was associated with increased psoas net muscle area, in keeping with other studies reporting a reduction in psoas muscle area among patients experiencing LBP [[20], [21], [22]].

Several studies have consistently demonstrated the multifidus muscle atrophy [23], especially in patients with chronic LBP [24,25]. In the present study we have not found significant results for the MRI parameters of paravertebral muscles status while the net area of psoas muscle showed a significant relationship with LBP. On this respect, a study by Elliott et al. [26] demonstrated that in patients with persistent pain related to a whiplash-associated disorder, the increase in cervical muscle CSA was due to fat infiltration, emphasizing the importance of qualitative muscle assessment. Even during remission of LBP, as previously demonstrated [13], a diffuse increase in fat infiltration of the lumbar muscles persisted without alterations in the lumbar muscle area. In the study conducted by Mengiardi et al. [27] MR spectroscopy quantification confirmed a significant increase in multifidus intramuscular fat among patients with chronic LBP compared to asymptomatic volunteers. These studies, in line with our findings, underlined the importance of evaluating the intramuscular fat infiltration and suggesting net muscular area as a fundamental parameter explaining non-invasively and in-vivo muscles quantity and quality.

In our study, the variable of disk degeneration showed no significant relationship with LBP at none of the lumbar levels assessed. Disk degeneration is believed to be the first step leading to spinal degeneration, it is highly prevalent in asymptomatic individuals, especially those with natural disk aging [8], with the last two lumbar levels being more susceptible [28]. The negative predictive value of typical signs for disk degeneration (such as loss of disk height, low T2-weighted intensity, and bulging disk) is reported to be high, while their positive predictive value and specificity remain low even when using grades 3 and 4 of the Pfirrmann classification [[29], [30], [31]]. These results suggest that although the relationship between disk degeneration and LBP is likely to be highly variable, possibly accounting for results of the present study. Indeed, a correlation between disk degeneration and paraspinal muscles’ increased fat infiltration has been reported, suggesting that these two entities might be linked to each other in the context of a "whole organ" degenerative disease [[29], [30], [31], [32]].

Interestingly, Ploumis and coworkers reported statistically significant unilateral atrophy of multifidus, erector spinae, quadratus lumborum, and psoas muscles on the symptomatic side of patients with longstanding unilateral back pain due to monosegmental degenerative disk disease [33]. These results underline and reinforce our findings on the importance of muscle status and the close interplay between disk degeneration, LBP and atrophy of the muscles as structures working together to warrant the correct spine biomechanics. On this respect, it should be emphasized that in this study we have not found a significant association between LBP and many of the variables explored, such as Pfirmann grade. These apparently conflicting results underline, once again, the complex multifaceted and multifactorial nature of LBP, which also carries variabilities due to a subjective tolerance threshold as reported by individual patients.

The present study also found a significant relationship between LBP and increased BMI while non-significant results for fat thickness and the presence/absence of subcutaneous edema. High BMI is a known risk factor for LBP and is also associated to subcutaneous edema [34]. In the present study, the relatively young age of the patients included (i.e., median 48.5 years) might partially account for this negative result on lumbar edema which might be more likely associated to longstanding LBP in patients with more advanced age.

The statistically significant relationships observed among VAS, BMI, and MRI-derived psoas cross-sectional area are intriguing. To enhance the clinical relevance, our study suggests potential applications, such as personalized weight loss physiotherapy strategies for workers with LBP. Utilizing MRI-derived metrics at baseline and through follow-up assessments could not only gage intervention efficacy but also facilitate adaptive changes, offering a tailored approach for improved patient outcomes. Thus, improving psoas-focused physiotherapy alongside weight management for LBP patients could be a promising avenue for personalized treatment with potentially improved outcomes.

There are limitations to consider in this study. First, the study design was retrospective, which may introduce inherent biases and limitations in data collection. Additionally, the study was conducted in a single center, which could limit the generalizability of the findings to other populations. Moreover, the sample size was relatively small. The study relied on self-reported measures, such as the VAS, for pain intensity, which may be subjective and influenced by individual interpretation. However, this is a quantitative indicator of LBP, still very useful to discriminate patients according to the degree of pain. Last, the relatively high proportion of females in the severe VAS group raises questions about gender's impact on muscle mass and its connection to LBP. On this respect, it should be noted that this difference, which emerged from descriptive statistics, is not statistically significant when assessed through bivariate analysis (Table 1). Further research with larger sample size is needed to validate these findings, better understand the role of muscles on LBP, and the relevance of gender-related variations in muscle composition to LBP. Moreover, future prospective studies could incorporate both self-reported and objective assessments of LBP. This would allow for a more comprehensive evaluation of the factors contributing to LBP. Indeed, it would be interesting to disentangle the cause-and-effect relationship by tracking individuals over time, monitoring changes in psoas size and LBP levels to provide a clearer picture of how they influence each other.

## Conclusions

In conclusion, we found a significant relationship between LBP intensity and psoas net muscle area or high BMI. These results highlight the importance of psoas muscle to stabilize the lumbar spine and suggest a protective role of a greater area of this muscle against LBP. The findings could contribute to future studies and improve treatment options in patients with LBP, possibly reducing the impact on disability, quality of life and the socioeconomical burden.

## Declaration of competing interest

The authors declare that they have no conflict of interest.
